# Challenges and Perspectives in Bridging In- and Outpatient Sectors: The Implementation of Two Alternative Models of Care and Their Effect on the Average Length of Stay

**DOI:** 10.3389/fpsyt.2017.00196

**Published:** 2017-10-05

**Authors:** Alexandre Wullschleger, Werner Wosniok, Jürgen Timm, Martin Heinze

**Affiliations:** ^1^Hochschulklinik für Psychiatrie und Psychotherapie der Medizinischen Hochschule Brandenburg, Immanuel Klinik Rüdersdorf, Rüdersdorf, Germany; ^2^Kompetenzzentrum für klinische Studien, Universität Bremen, Bremen, Germany

**Keywords:** integrated care, psychiatric care, outpatient treatment, care models, duration of stay

## Abstract

New models of care aimed at reinforcing the outpatient sector have been introduced in Germany over the last few years. Initially, a subscription-based model (“integrated care”) was introduced in 2012 in the Immanuel Klinik Rüdersdorf, wherein patients had to actively subscribe to the integrated care program. This integrated care model was replaced after 2 years by a subscription-free “model project,” in which all patients insured by the contracting insurance company took part in the program. Data showed that the introduction of the integrated care program in the inpatient setting led to an increase of the average length of stay in this group. The switch to the model project corrected this unwanted effect but failed in significantly decreasing the average length of stay when compared to standard care. However, both the integrated care program and model project succeeded in reducing the length of stay in the day care setting. When adjusting for the sex and diagnosis proportions of each year, it was shown that diagnosis strongly influenced the average length of stay in both settings, whereas sex only slightly influenced the duration of stay in the inpatient setting. Thus, in spite of strong financial and clinical incentives, the introduction of the model project couldn’t fulfill its primary purpose of shifting resources from the inpatient to the outpatient setting in the initial years. Possible explanations, including struggle against long-established traditions and reluctance to change, are discussed.

## Introduction

In the past few years, Germany has experimented with new models of care in order to repair the known deficits of the German psychiatric care system. Namely, it sought to counter the unbalanced allocation of resources, and the lack of interface management between the in- and outpatient sectors. These deficits have already been described in depth in previous studies and reports (1, 2).

The first initiative took the form of regional budgets. Regional budgets are financing models that are based on the cooperation between regional health care providers and all health insurance companies involved. In this model, a global annual budget is allocated to hospitals by the insurance companies to finance psychiatric care. The use of in- and outpatient resources is financed by this budget under the sole responsibility of the care providers, who make all decisions regarding their allocation. This annual budget creates a strong financial incentive to reduce the use of inpatient care and develop more comprehensive offerings in the outpatient sector. Such a project was established for the first time 10 years ago in the region of Steinburg. Meanwhile, other projects emerged, particularly in Schleswig-Holstein, but also in Nordhausen/Thüringen. However, the legal basis of the regional budgets [§26 of the “Bundespflegesatzverordnung” (German National Hospital Rate Ordinance)], set important barriers to their implementation, notably that all health insurance companies must agree to the regional budget.

The so-called integrated care (legally governed by the §140 a SGB V), presents itself as an alternative to regional budgets. In this model, the care provider receives annually a fix amount of money per patient subscribing to the program. It can be implemented in the community as well as in hospitals without the participation of all insurance companies, since it is based on cooperation agreements between a care provider and a single insurance company. Examples of integrated care models are the projects located at the University Hospital Hamburg/Eppendorf, in Munich (Klinikum München-Ost) as well as home treatment models in Krefeld und Frankfurt (3).

Data regarding the effects of integrated care projects and regional budgets on clinical and financial outcomes are, to date, scarce. Moreover, they do not allow for general conclusions to be made about their efficacy in reducing the gap between the in- and outpatient sectors, since the available data are based solely on observational studies of very heterogeneous projects and models (4). After 5 years, the accompanying research of the University of Leipzig showed that the use of inpatient resources within the regional budget in Steinburg was considerably reduced. The rates of day- and outpatient care were concomitantly increased (5–8). In Munich, the average length of stay decreased since the implementation of the new integrated care model and patients reported a high level of satisfaction, although the implementation process was not free of difficulties (9). In Hamburg, a new model that focused on severely ill patients (F2x und F3x ICD-10 diagnostic codes) contributed to an increase in the outpatient contacts, a decrease of the average inpatient length of stay, and higher patient satisfaction by maintaining cost-effectiveness (10–12). In Krefeld, a new home treatment-based implemented model proved to be effective in increasing the satisfaction of patients and their next of kin, while preserving a constant quality of care when compared to standard inpatient care (13, 14). Finally, the integrated care project of the public insurance provider DAK-Krankenkasse has been implemented in four regions in Germany. It is based on a close cooperation with psychiatrists in private practices and has led to a significant reduction of inpatient length of stay, as an observational evaluation study showed (15).

The new patient-linked remuneration system of the integrated care models should offer a more flexible form of care. In this model, the service providers carry the responsibility for the allocation of resources and hence bear all the financial risks. As in the case of regional budgets, this should be a powerful financial incentive to reduce the costs of the inpatient sector and to develop more comprehensive outpatient care including assertive community treatment (ACT) and home treatment (16). A stronger and more dynamic cooperation with the outpatient sector should allow patients to be discharged earlier and thus decrease the average inpatient length of stay.

A potential problem of the integrated care model is that patients must actively subscribe to it, with the risk that many severely ill patients, who should primarily benefit from such a program, do not get to subscribe to it. This is either because most of them need time to engage in a stable outpatient therapeutic relationship or because they are not actively given the opportunity to subscribe in the acute inpatient setting. Data regarding this issue are controversial: in a previous work, we confirmed this hypothesis by showing low subscription rate by patients with a F2 diagnosis (17), whereas another study showed higher subscription rates of patients with a F2/F31 diagnosis when compared to other diagnostic groups (18).

To avoid this potential negative effect, the legislator introduced another legal basis governing the development of new models of integrated care. The §64b SGB V stipulates that at least one so-called “model project” consisting in an agreement between care providers (e.g., hospitals) and an insurance company should be implemented in every federal state. All patients insured by the contracting insurance company benefit from the program without active subscription, thus allowing the most severely ill patients to be part of such a program. This should help to reduce the duration of their hospital stays and to transfer them effectively in the outpatient care.

An integrated care model was implemented in the Immanuel Klinik Rüdersdorf in 2012 on the basis of the described subscription model (§140a) in cooperation with one, and later two, insurance providers (Techniker Krankenkasse and Barmer GEK). After 2 years, the model switched to the new subscription-free model project (§64b) to counter the described negative effects of the subscription model as well as the increase of the lengths of stay observed in our hospital in 2013.

The present study aims at evaluating if the switch to the model project led to a shortening of the average length of stay when compared to the integrated care program and to standard care.

We thus here analyze and compare the average lengths of stay of patients of these three groups (integrated care, model project, standard care).

## Materials and Methods

The data analysis is based on patient’s data available in the hospital information system (here SAP) that have been analyzed using the associated software. Patients admitted to the hospital over the course of 2013 and 2014 were divided in three groups: standard care (2013 and 2014), integrated care after §140a (2013), and model project after §64b (2014). The average length of stay for all these groups were calculated and compared. Patients insured by the cooperating insurance company actively subscribed to the integrated care model in 2013. In 2014, all patients insured by both cooperating companies were included in the model project, without the need to subscribe. Patients admitted at the end of 2013 remained under the regime of integrated care (§140a). Their calculated length of stay has been taken into account for 2013. Length of stay of patients admitted in the end of 2014 and discharged in 2015 has been taken into account for 2014. No change from one model to another occurred.

To describe and analyze the influence of confounding factors, a multifactorial analysis of the length of stay was made including sex, age, and diagnosis (after ICD-10) as potential explaining factors.

The statistical analysis was carried out using the SAS 9.4, TS1M319 package (19). For checking the equality of proportions, the asymptotic χ^2^ test (*n* ≥ 1,000) or Fisher’s exact test (*n* < 1,000) was used. Mean lengths of stay were compared by Student’s *t* test. For estimating the model effect on the length of stay while adjusting for effects of sex, age, or diagnosis group, a backward analysis of variance (ANOVA) was carried out. Starting model in all cases was in symbolic form: length of stay = model + year + diagnosis group + sex + age group + diagnosis group * model. The factor “model” has two values: standard treatment and non-standard treatment, where the latter means treatment within the integrated care program (in 2013) or treatment within the model project (in 2014). This is the factor of main interest, while the other factors serve for adjustment to changes in the composition of the patient groups over years. Type III sum of squares were used to assess the importance of factors. For all tests a *p* value ≤ 0.05 (α = 5%) was considered to signal a significant difference.

The present study was conducted solely on the basis of anonymized data retrieved from the hospital information system and didn’t imply the direct involvement of patients. Hence, it did not require the approbation of the local ethics committee.

## Results

### Demographic Characteristics of the Patients Sample

The patients’ demographic characteristics are summarized in Table 1.

**Table 1:** Patient counts by sex and age group.

Inpatient setting		2013		2014	
		Integrated	Standard	Model	Standard
	Total	119	1,514	375	1,179
	Women (%)	56 (47.0%)	693 (45.7%)	180 (48.0%)	477 (40.4%)
Age groups (%)	<18 years	–	1 (0.07%)	1 (0.3%)	–
	18–35 years	25 (21.0%)	389 (25.7%)	92 (24.5%)	253 (21.5%)
	36–55 years	62 (52.1%)	645 (42.6%)	189 (50.4%)	532 (45.1%)
	56–65 years	14 (11.7%)	249 (16.4%)	41 (10.9%)	189 (16.0%)
	>65 years	18 (15.1%)	230 (15.2%)	52 (13.9%)	205 (17.4%)

**Day care setting**		**2013**		**2014**	
		**Integrated**	**Standard**	**Model**	**Standard**

	Total	60	304	161	249
	Women (%)	38 (63.3%)	202 (66.4%)	105 (65.2%)	159 (63.8%)
Age groups (%)	<18 years	–	–	–	1 (0.4%)
	18–35 years	19 (31.7%)	111 (36.5%)	58 (36.0%)	80 (32.1%)
	36–55 years	29 (48.3%)	153 (50.3%)	80 (49.7%)	118 (47.4%)
	56–65 years	10 (16.7%)	38 (12.5%)	19 (11.8%)	42 (16.9%)
	>65 years	2 (3.3%)	2 (0.7%)	4 (2.5%)	8 (3.2%)

### Average Length of Stay

In 2013, the average length of stay of patients in inpatient setting who subscribed to the integrated care model was 26.8 days (*n* = 119). The length of stay of patients who did not take part in this program was 20.0 days (*n* = 1,514). This difference was shown to be statistically significant (*p* = 0.003). In 2014, the average length of stay of patients in the model project was 20.7 days (*n* = 375). Patients in the standard care group stayed in the hospital 19.5 days in average (*n* = 1,179, *p* = 0.397), thus showing no statistically significant difference. The comparison of the average lengths of stay between the integrated care group (2013) and the model project group (2014) showed a statistically significant reduction of the average length of stay (*p* = 0.017). These results are summarized in Table 2.

**Table 2:** Mean length of stay of inpatients by diagnosis group and sex.

Inpatient setting		2013		2014		*p* Value (integrated care vs. model project)
		Integrated	Standard	Model	Standard	
All diagnoses	*n*	119	1,514	375	1,179	
	L. of stay (d)	26.8	20.0	20.7	19.5	0.017
Male	*n*	63	821	195	702	
	L. of stay (d)	23.2	15.7	17.1	17.5	0.069
Female	*n*	56	693	180	477	
	L. of stay (d)	30.9	25.2	24.6	22.4	0.087
F00–F09	*n* (%)	1 (0.8%)	78 (5.2%)	16 (4.3%)	70 (5.9%)	
	L. of stay (d)	11.0	16.3	17.6	14.9	*–*
F10*–*F19	*n* (%)	28 (23.5%)	602 (39.8%)	135 (36.0%)	456 (38.7%)	
	L. of stay (d)	12.9	10.6	10.3	11.0	0.17
F20–F29	*n* (%)	17 (14.3%)	220 (14.5%)	46 (12.3%)	159 (13.5%)	
	L. of stay (d)	26.9	31.4	43.6	34.2	0.14
F30*–*F39	*n* (%)	67 (56.3%)	439 (29.0%)	129 (34.4%)	326 (27.7%)	
	L. of stay (d)	33.8	32.4	27.6	28.4	0.095
F40–F49	*n* (%)	5 (4.2%)	125 (8.3%)	42 (11.2%)	131 (11.1%)	
	L. of stay (d)	15.0	9.1	11.3	12.1	0.59
F60–F69	*n* (%)	–	28 (1.8%)	7 (1.9%)	19 (1.6%)	
	L. of stay (d)	–	9.3	6.7	15.1	–

*Indicated *p* values refer to *t* test results for comparing the mean lengths of stay under the integrated care program (2013) with the model project (2014)*.

In day care setting, the average duration of stay of patients who subscribed to the integrated care program in 2013 was 30.4 days (*n* = 60), whereas patients in the standard care group showed a significantly longer average length of stay [36.6 days (*n* = 304)] (*p* = 0.017). A similar difference could be shown for 2014 after the transition to the model project: patients in the model project showed shorter lengths of stay when compared to the standard care group [31.6 days (*n* = 161) vs. 35.9 days (*n* = 249)] (*p* = 0.008). No significant difference in the length of stay between the integrated care and model project could be shown (*p* = 0.901). These results are summarized in Table 3.

**Table 3:** Mean length of stay of day care patients by diagnosis group.

Day care setting		2013		2014		*p* Value (integrated care vs. model project)
		Integrated	Standard	Model	Standard	
All diagnoses	*n*	60	304	161	249	
	L. of stay (d)	30.4	36.8	31.6	35.9	0.90
F10–F19	*n* (%)	–	1 (0.3%)	4 (2.5%)	7 (2.8%)	
	L. of stay (d)	–	10.0	6.8	21.7	–
F20–F29	*n* (%)	4 (6.7%)	15 (4.9%)	11 (6.8%)	17 (6.8%)	
	L. of stay (d)	26.0	36.9	27.6	40.2	0.88
F30–F39	*n* (%)	53 (88.3%)	251 (82.6%)	119 (73.9%)	189 (75.9%)	
	L. of stay (d)	31.0	37.1	32.3	37.7	0.97
F40–F48	*n* (%)	1 (1.7%)	24 (7.9%)	16 (9.9%)	25 (10.0%)	
	L. of stay (d)	12.0	30.8	29.9	28.8	–
F60–F69	*n* (%)	2 (3.3%)	13 (4.3%)	11 (6.8%)	11 (4.4%)	
	L. of stay (d)	33.0	40.2	39.8	23.9	0.74

*p Values refer to *t* test results for comparing the mean lengths of stay under the integrated care program (2013) with the model project (2014)*.

### Diagnosis and Length of Stay

The average lengths of stay in each diagnostic group (after ICD-10) in both inpatient and day care setting are summarized in Tables 2 and 3. No statistically significant difference regarding diagnostic groups could be shown between 2013 and 2014 for patients in the integrated care and model project in both inpatient and day care setting.

### Analysis of Variance

**Figure 1:**
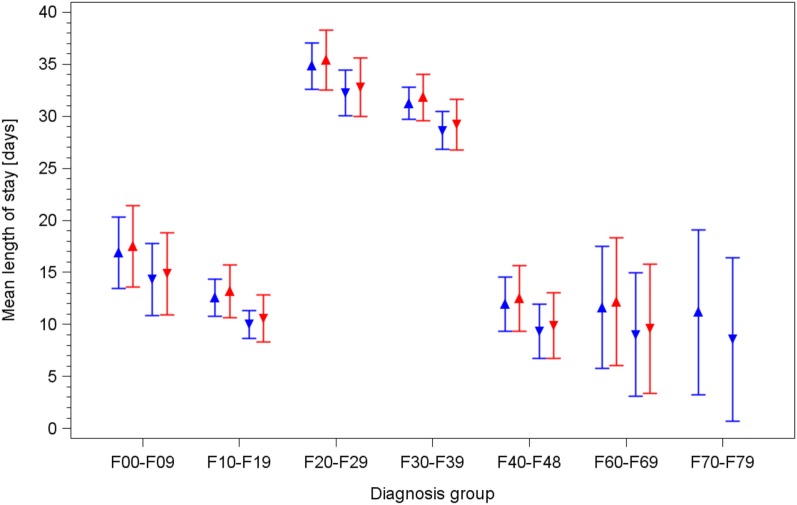
Mean duration of stay for inpatients in both standard care and integrated care/model project groups, by diagnosis and sex, estimated by the final model of backward analysis of variance. Upward triangles: females; downward triangles: males. Blue lines/symbols: standard care; red lines/symbols: integrated care/model project.

In the inpatient setting, the ANOVA for the factors potentially influencing the average length of stay in both standard and integrated care/model project groups (type of model, age, sex, diagnosis, and year) showed that it strongly depends on diagnosis and sex. Patients of the F2 and F3 groups showed significantly longer lengths of stay compared with the other diagnosis groups. Male patients stayed on average 2.6 days shorter than female patients. Participation to the integrated care program or model project only slightly influenced (*p* = 0.594) the length of stay. No influence of age could be shown. The integrated care program and model project showed no diagnosis-specific effect compared to the subscription program on the average length of stay. These results are shown in Table 4. Estimated durations of stay for the various subgroups are shown in Figure 1, together with their 95% confidence intervals.

**Table 4:** Final results of backward analysis of variance for inpatients.

Source	DF	Type III SS	Mean square	F value	Pr > F
Type of treatment (standard or inscription/model)	1	135.5	135.5	0.28	0.5944
Diagnosis group	6	285,842	47,640	99.67	<0.0001
Sex	1	4,576	4,576	9.57	0.0020

*Estimated durations of stay for the various subgroups are shown in Figure **1***.

**Figure 2:**
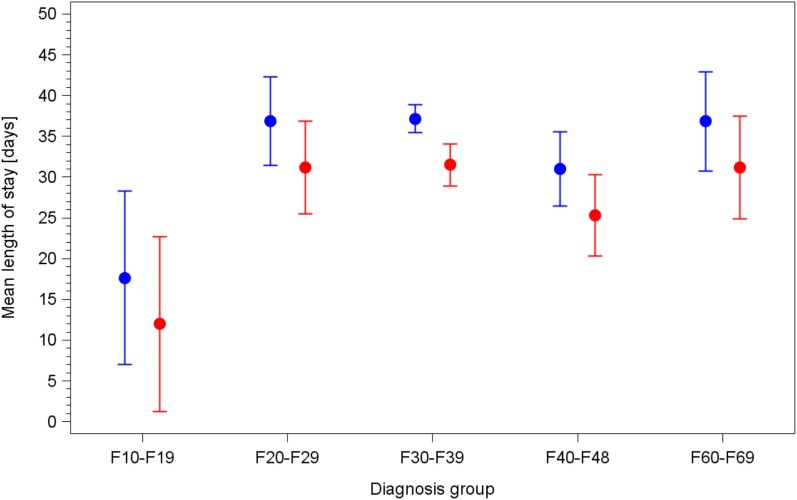
Mean duration of stay for day care patients in both standard care and integrated care/model project groups, by diagnosis group, estimated by the final model of backwards analysis of variance. Blue lines/circles: standard care; red lines/circles: integrated care/model project.

In day care setting, the ANOVA showed that the average length of stay strongly and significantly depended on the diagnosis and on participation to the integrated care/model project, with patients in this group staying 5.7 days shorter on average. No specific effect of age, sex, or an interaction of diagnosis and type of model could be shown. These results are shown in Table 5. Estimated durations of stay for all relevant subgroups are shown in Figure 2, together with their 95% confidence intervals.

**Table 5:** Final results of backward analysis of variance for day care patients.

Source	DF	Type III SS	Mean square	*F* value	Pr > *F*
Type of treatment (standard or inscription/model)	1	5,052	5,052	14.52	0.0001
Diagnosis group	4	6,495	1,624	4.67	0.0010

*Estimated durations of stay for the various subgroups are shown in Figure **2***.

## Discussion

The introduction of a subscription-based integrated care model was supposed to lead to shorter average lengths of stay. Such an effect in an inpatient setting could not be observed in the present study. On the contrary, the average length of stay in this setting rose, against expectations, whereas they decreased as expected in day care setting.

This result was obviously linked to the negative effect of the subscription model and the repartition of diagnoses it led to, as shown in a previous work in which possible explanations are discussed (17). These include a stronger desire for more comprehensive and intensive treatments by patients with affective disorders; a difficulty to reach many patients who stay only a few days in the hospital, thus making an active subscription to the program difficult; and the fact that many patients with an addictive disorder only completed short treatment courses without being willing to engage in longer and comprehensive outpatient treatments. Thus we expected to counter this effect after switching to the newly introduced subscription-free model project. The hypothesis was that this switch would correct the observed shift in the distribution of diagnostic groups in the integrated care program and thus lead to a shortening of the average length of stay when compared to the integrated care program. Furthermore, we hoped that the model project could show its expected effect on the duration of inpatient stay compared to standard care.

The results partly confirmed our hypothesis in the inpatient setting. There, the switch to the subscription-free model project led to a statistically significant shortening of the average length of stay when compared to the old integrated care program but not when compared to the standard care group. ANOVA results showed that the mix of diagnoses plays a central role in the average length of stay with patients of the F2 and F3 group showing longer duration of stay. An analysis of the repartition of the diagnostic groups in both the integrated care program and the model project showed that patients with a F3 diagnosis were overrepresented and patients with a F1 diagnosis underrepresented in the integrated care program. The overrepresentation of patients of the F3 group led to an increase in the average length of stay that could then be countered with the new model project. ANOVA results for the inpatient setting showed age did not affect the average length of stay. Surprisingly, however, sex was shown to influence the average length of stay, with male patients having a slightly shorter stay in the hospital than women. This is explained by the higher rate of substance abuse disorders among male patients (2013: 481 male patients vs. 149 female patients; 2014: 452 male patients vs. 139 female patients), which, in our experience, often leads to early discharge due to reluctance to engage in long-term treatment.

In contrast to this, the results for the day care setting showed no difference in the average lengths of stay between the integrated care program and the model project. In this setting, ANOVA showed an effect of diagnosis on the length of stay in a similar way as in the inpatient setting. It also showed that both new models of care also played a role for the length of stay, but neither age nor sex. A possible explanation for the lack of effect from the switch to the model project in the day care setting can be found when analyzing the diagnostic repartition of patients in both groups: the introduction of the subscription-free program did not lead to a shift in the diagnostic repartition, as was the case in the inpatient setting. The longer duration of stay, the traditional greater focus on long-term rehabilitation in a day care setting, and the persistent overrepresentation of affective disorders also explain that needing to subscribe to the integrated care program did not represent an obstacle in this setting.

However, when compared to standard care, both new models of care led to a reduction in the average length of stay that could not be shown in the inpatient setting. A possible explanation could lie in the particular configuration of our hospital. The day care department is located in the same buildings as the outpatient department, and both are located outside of the main hospital building where the inpatient sector is located. This proximity surely reinforces the cooperation and synergies between day care and outpatient sectors and thus promotes a faster transition in the outpatient care. This reduction of the average length of stay in the integrated care program and model project is in line with the results of previous studies evaluating the effect of such programs on the average duration of stay (9, 15).

The shift of psychiatric care resources from the in- to the outpatient sector, and the reduction of average length of stay are crucial and have been addressed in many countries over the last years. The newly introduced model projects could represent a great opportunity to deal with this issue. The shift of the financial risk from the insurance companies to the service providers requires the development of more comprehensive outpatient care, including home treatment, ACT, and a reinforced cooperation between the in- and outpatient sectors in order to reduce the use of inpatient resources and efficiently reduce the average length of stay. This would be in line with orientations wished by service providers, patients and their relatives. In spite of these strong incentives, in the year following the introduction of the new model project the expected effect could only be marginally observed in our study.

Surely the aforementioned geographical specificities of the hospital or the lack of a proper mobile home treatment/ACT unit combined with the great distances between clients in the region played a role in preventing the model project from having its full impact on the duration of stay. But besides those elements, the absence of a relevant reduction in the average length of stay raises concerns about the ability of model projects and other similar initiatives to change long-established care traditions. To date, the organization of psychiatric hospitals and wards has been directed at offering comprehensive inpatient care, which was also the main financial resource of institutions. In many places, the development of new models of outpatient treatment, such as ACT/home treatment, has been neglected. Staff members are often reluctant to engage in new models of care that imply such a profound change in the definition and practice of inpatient psychiatric care. Inpatient treatment devolves into intensive crisis management, often leaving symptoms remission and recovery to the outpatient sector. Such a change is often seen by staff members as a challenge to their ability to take care of acutely ill patients and to offer them comprehensive treatment. Such difficulties have already been described in the implementation process of new models of care (20). Hence, the introduction of such a model should be seen as a long-term process involving profound changes in traditions and routines.

Also, staff members in public institutions are often not used to consider economic factors in their everyday practice, what represents in case of new initiatives such as model projects an obstacle to their full implementation. Model projects require the full commitment of all staff members in managing the limited financial resources allowed by the insurance company and allocating them preferentially to the outpatient sector.

In conclusion, model projects constitute a possible way of bridging in- and outpatient care for all patient categories, particularly the most severely ill. However, their ability to efficiently reduce the average length of stay and hence to strengthen and develop outpatient care still needs to be proven. The discussed obstacles to their full implementation should be addressed by reinforcing the commitment of all staff members and by supporting the profound changes of structures and practices they imply. Only then can reluctance and long-established routines be overcome.

One of the most important limitations of the present study is the lack of outpatient data. Unfortunately, these are not available through the hospital information system, thus rendering their analysis impossible. Such data would however be of great interest and should be taken into consideration in further studies aimed at evaluating the implementation of new models of care and their effect on the average duration of stay.

## Author Contributions

AW contributed to the conception of the research article, data collection and processing, results discussion and manuscript redaction. WW and JT contributed to the statistical analysis of the data and their presentation. MH contributed to the conception of the article, discussion of the results, and manuscript redaction and supervised the whole process.

## Conflict of Interest Statement

The authors declare that the research was conducted in the absence of any commercial or financial relationships that could be construed as a potential conflict of interest.
